# Evaluation of three 3ABC ELISAs for foot-and-mouth disease non-structural antibodies using latent class analysis

**DOI:** 10.1186/1746-6148-2-30

**Published:** 2006-10-16

**Authors:** Barend M deC Bronsvoort, Nils Toft, Ingrid E Bergmann, Karl-Johan Sørensen, John Anderson, Viviane Malirat, Vincent N Tanya, Kenton L Morgan

**Affiliations:** 1 Centre for Tropical Veterinary Medicine, University of Edinburgh, Easter Bush Veterinary Centre, Roslin, Midlothian, EH25 9RG, UK; 2 Dept. Veterinary Clinical Sciences and Animal Husbandry, University of Liverpool, Leahurst, Neston, Wirral, CH64 7TE, UK; 3 Institute of Agricultural Research for Development, Regional Centre of Wakwa, B.P. 65, Ngaoundere, Cameroon; 4 Institute of Animal Health, Ash Road, Pirbright, Woking, Surrey, GU24 0NF, UK; 5 Danish Institute for Food and Veterinary Research, Virology Department, Lindholm, DK4771 Kalvehave, Denmark; 6 Dept. of Large Animal Sciences, The Royal Veterinary and Agricultural University, Groennegaardsvej 8, DK-1870 Frederiksberg C, Denmark; 7 Pan-American Foot-and-Mouth Disease Center (PAHO/WHO), Rio de Janeiro, Brazil; 8 Ministry of Scientific Research and Innovation, P.O. Box 1457, Yaounde, Cameroon

## Abstract

**Background:**

Foot-and-mouth disease (FMD) is a highly contagious viral disease of even-toed ungulates. Serological diagnosis/surveillance of FMD presents several problems as there are seven serotypes worldwide and in the event of vaccination it may be necessary to be able to identify FMD infected/exposed animals irrespective of their vaccination status. The recent development of non-structural 3ABC protein (NSP) ELISA tests has greatly advanced sero-diagnosis/surveillance as these tests detect exposure to live virus for any of the seven serotypes of FMD, even in vaccinated populations. This paper analyses the performance of three NSP tests using a Bayesian formulation of the Hui-Walter latent class model to estimate test sensitivity and specificity in the absence of a "gold-standard" test, using sera from a well described cattle population in Cameroon with endemic FMD.

**Results:**

The analysis found a high sensitivity and specificity for both the Danish C-ELISA and the World Organisation for Animal Health (O.I.E.) recommended South American I-ELISA. However, the commercial CHEKIT kit, though having high specificity, has very low sensitivity. The results of the study suggests that for NSP ELISAs, latent class models are a useful alternative to the traditional approach of evaluating diagnostic tests against a known "gold-standard" test as imperfections in the "gold-standard" may give biased test characteristics.

**Conclusion:**

This study demonstrates that when applied to naturally infected zebu cattle managed under extensive rangeland conditions, the FMD ELISAs may not give the same parameter estimates as those generated from experimental studies. The Bayesian approach allows for full posterior probabilities and capture of the uncertainty in the estimates. The implications of an imperfect specificity are important for the design and interpretation of sero-surveillance data and may result in excessive numbers of false positives in low prevalence situations unless a follow-up confirmatory test such as the enzyme linked immunoelectrotransfer blot (EITB) is used.

## Background

Foot-and-mouth disease (FMD) is a highly contagious viral disease of even-toed ungulates caused by *Foot-and-mouth disease virus *(FMDV), which is a member of the genus *Aphthovirus *and the family *Picornviridae *[1]. A number of non-structural protein (NSP) immunoassays, designed to detect aphthovirus-specific antibodies to replicating virus in animals irrespective of vaccination status or for screening multiple-serotype infected/exposed animals, have been under development in recent years [2-9].

The OIE Reference Laboratory for the Americas, PANAFTOSA/PAHO/WHO, has developed and validated a surveillance tool, to accompany the progress of eradication campaigns in South America, based on a screening NSP ELISA (the I-ELISA 3ABC), followed by an enzyme linked immunoelectotransfer blot test (EITB) to clear up false positive reactions [10]. Because vaccination prevents clinical disease but does not necessarily prevent infection or persistence [11-13], it is useful to be able to identify animals which are infected or have been exposed to live virus irrespective of their vaccination status. The NSP tests offer an alternative to the virus neutralization test (VNT) [14] and liquid phase blocking ELISA (LPBE) [15-17]. The VNT and LPBE detect antibodies to FMDV structural proteins and require separate testing for each of the seven serotypes of FMDV and for some subtypes. These tests are time consuming to perform, require virus containment facilities and cannot differentiate vaccinated from convalescing animals [18]. Furthermore the VNT may not be an appropriate comparison for NSP ELISAs since antibodies to the structural proteins are detected slightly sooner and for longer than NSP antibodies [19].

The need for a mass screening test for use in vaccinated populations to detect circulating infection gained impetus in Europe with the outbreak of FMD in the UK in 2001. The mass slaughter of millions of sheep and cattle caused public outrage and has added a political element to the arguments that vaccination should be used in future FMD outbreaks. The United Kingdom came close to vaccinating dairy herds in Cumbria prior to turn-out of cattle in the spring of 2001 but eventually opted not to amid fears of prolonging trade bans and consumer resistance to drinking milk from vaccinated cattle [20]. EU regulations have since been modified to allow for the emergency use of vaccination (EU Council Directive 85/511/EEC). The World Organization for Animal Health (O.I.E.) trade regulations at the time required 12 months of disease freedom following the end of emergency vaccination before a country could be declared free. However, in 2004 the regulations were altered and countries can now regain 'FMD free' status six months after the end of vaccination providing they have carried out surveillance with an NSP test (Article 2.2.10.7, O.I.E. Terrestrial Animal Health Code, 2004). This is three months later than could be achieved following a slaughter policy without vaccination.

In order to design and implement an effective vaccination program and subsequent sero-surveillance, it is necessary to know the properties of the test(s) intended to be used. The properties of a diagnostic test are often described using the sensitivity (Se), i.e. the proportion of true positives that the test identifies and the specificity (Sp), i.e. the proportion of true negatives that the test identifies. When available, these parameters are estimated using animals with known infectious status. For NSP tests the values have been obtained using controlled experimental and field models [8,10,21-23]. Alternatively, studies can be carried out to compare the diagnostic sensitivity and specificity of an assay against a standard of comparison i.e. a "gold standard" test.

Previously some of the authors compared two FMD 3ABC ELISAs using the VNT as the "gold-standard" [24], i.e. it was assumed that the Se and Sp of the VNT were both 100%. However, there might be reasons to question the justification of using the VNT as a "gold-standard". It appears, that reliable estimates of the duration of the different antibody responses in cattle are lacking. Thus, there might be justification in disregarding the VNT and compare the NSP tests to each other without assuming that the true status of the animal is known (i.e. the disease status is latent). The latent class approach does not assume any of the tests is a "gold standard" [25-27]. This paper follows on from our previous evaluation of two NSP tests using a conventional approach with the VNT as a "gold-standard" [24].

The objective of the current study was to estimate sensitivity and specificity of two previously studied NSP tests, the CHEKIT-FMD-3ABC bo-ov (CHEKIT) ELISA (Bommeli Diagnostics/Intervet) and the 3ABC blocking ELISA (C-ELISA) developed in Denmark, as well as the O.I.E. index NSP test, the I-ELISA 3ABC developed by PANAFTOSA [10] without assuming that a 'gold standard' was available. The test properties were estimated for each of the three tests in a cattle population from Cameroon where serotypes O, A and SAT2 were known to be circulating and where at the time of the original study in 2000, there was no vaccination [28,29]. The analysis used a Bayesian formulation of the Hui-Walter latent class model for test evaluation in the absence of a "gold-standard" [26]. Since this work was carried out the CHEKIT-FMD-3ABC bo-ov has been reformulated as a mark II kit and the Danish C-ELISA has been reformulated as a commercial kit and marketed under the name Ceditest^® ^FMDV-NS (Cedi Diagnostics B.V.).

## Results and discussion

Based on the data given in Table 1, the Se and Sp of the three tests were estimated using the full set of adult and juvenile animals in the five administrative Divisions (subpopulations). To facilitate the discussion of the underlying assumptions of the latent class model, the test parameters were estimated both by combining all three tests and then by using only two tests at a time. The results of the analysis on the full data set are given in Table 3. The parameter estimates based on the data from all three tests gives high posterior mean estimates for the Se for both the C-ELISA (96.9%) and the I-ELISA (97.1%) but a very low posterior mean estimate for the CHEKIT (33.2%). Conversely, the posterior mean Sp is very high for the CHEKIT (99.0%) but lower for both of the other tests (90.9% and 88.8% respectively). However the posterior 95% credibility interval (PCI) appear to overlap and there is little evidence of a significant shift in the estimates though the PCI are wider.

The results for the sub-set of juvenile animals (8–24 months old) which use the data from Table 2 are given in Table 4. Based on this smaller data set and using the three tests combined, the CHEKIT posterior mean estimate shows a small change with a slight reduction in the Se (26.1%). The C-ELISA also has a lower Se posterior mean estimate (92.6%) but the Sp is conversely slightly higher (96.1%). A similar shift is seen with the I-ELISA (92.3% and 93.7% respectively).

The key issue in traditional test parameter estimation is the availability of a "gold-standard" test, i.e. a test whose outcome can be directly interpreted as presence or absence of disease/infection. Quite often however, the need for such a test seems to determine the definition of the disease and this may introduce selection bias in the test evaluation [30] or force a disease definition, which does not necessarily reflect the essential properties of the disease, but merely the detectable properties. The estimates for the relative Se and Sp of the C-ELISA test when compared to the VNT as a "gold standard" for this cattle population were 71% and 90% respectively [24]. The new best estimate of 96.9% for the Se of the C-ELISA is much higher, although the estimate for the Sp did not change (90.0%). This difference is likely to reflect the problem of defining what a seropositive animal means. Using the VNT as a "gold standard" results in animals exposed to FMD virus up to several years previously [22,31-33], being classified as seropositive due to the long half-life on neutralising antibodies [32]. By contrast, NSP antibodies do not appear to persist for more than 6–12 months [6,22] though there seems to be much variation in these estimates [34] and so a different set of animals will be classified as seropositive by an NSP test. This difference in test classification using a poor "gold-standard" could result in the comparative test (in this situation the NSP test) appearing to perform poorly. However, from a disease control and a decision maker's point of view, what is important is evidence of recent exposure to an FMD virus or continuing sub-clinical disease post-vaccination which the NSP tests are directed at.

Using a latent class analysis circumvents the need for an available "gold-standard" test hence providing an alternative to the classical analyses where the problems with selection bias or improper disease definitions. However, there are explicit and implicit assumptions of the latent class analysis, which require that the results of the analysis are scrutinized. Specifically, there is a need to explore the definition of the disease imposed by applying a set of tests. Essentially the definition of disease in a latent class analysis can be perceived as whatever the tests agreed upon. The impact of this is that the definition of disease might depend on the subset of tests used in the analysis. This is very clearly illustrated in Toft *et al. *[35], where the choice of tests in the latent class analysis dramatically changes the properties of the tests, due to a changed perception of what constitutes a "diseased" individual. The problem is, essentially, that a test result rarely is a direct measure of what we are looking for in the test subject. An example is the use of antibody tests which may detect past exposure rather than current infection with the agent.

In order to explore how the choice of tests might impact the definition of disease in this cattle population, we did a pair-wise comparison of the 3ABC ELISA tests to compare the test properties as well as the estimated prevalences when using all three tests opposed to any combination of just two tests (Tables 3). Given the uncertainty associated with the estimates, there are no discernable differences between the estimates of Se, Sp and prevalence across the combinations of test. This leads us to conclude that the disease definition remains the same regardless of which tests are used for the analysis.

However, for the juvenile sub data set (Table 4), there seems to be some inconsistency between estimates when doing pair-wise comparisons, most noticeably in the estimate of the Se of the CHEKIT ELISA. Comparing the scenario with all three tests to the C-ELISA verses the I-ELISA there are no relevant differences, but when either of these two tests are evaluated alone against the CHEKIT ELISA the estimates seem to shift towards something that indicates a changed perception of what constitutes a "diseased" case. However, it is the author's experience that this shift might also be explained by the combination of the poor Se of the CHEKIT ELISA and the relatively small sample size.

The assumption of conditional independence between the tests given disease status is vital to the presented analysis. The disease status defined by three NSP ELISAs for FMD virus must be considered a measure of the presence of NSP antibodies. Conditional on such a 'disease status' there is no reason to assume that the three tests are not conditionally independent. It is possible to assess pair-wise conditional dependence, i.e. assuming that one tests is conditionally independent given disease status of the other two potentially dependent tests. However, a model assuming that all three tests are conditional dependent given disease status is unidentifiable and can as such not be evaluated without specifying informative priors. Furthermore, it would not make sense to assume such a correlation structure as it would merely imply that none of the tests were measuring what we are looking for. For completeness, we compared the three models allowing for pair-wise conditional dependence to the model assuming conditional independence between tests given disease status using the Deviance Information Criteria (DIC) [36]. The DIC did not suggest that any of the models allowing conditional dependence between two tests given disease status were to be preferred over the model assuming conditional independence between the three tests given disease status.

The prevalence estimates from this analysis follow a similar pattern across the five Divisions to the herdsmen reported outbreaks for the 12 months prior to the interview based on a structured questionnaire. The herdsman reported estimates were 77%, 57%, 37%, 44% and 73% for the Vina, Mbere, Djerem, Mayo Banyo and Faro et Deo Divisions respectively [37,38]. The model assumes that the tests perform the same in all the sub-populations (i.e. Divisions) and that only the true prevalence differs. These Divisions were the obvious way to divide the population but others could have been used. The effects of using different ways to divide the population were not explored. The prevalence estimates based on the juveniles only were significantly lower than for the full population sample. This would be expected since only relatively recent exposure is captured in this subpopulation.

The estimates of Se and Sp for the NSP tests presented here are lower than those obtained from experimental and field models in South American livestock populations for the I-ELISA [9,10,21] as well as for the C-ELISA and CHEKIT tests (unpublished results). Studies using sera panels from FAO have also suggested very high parameter estimates of 98% Se and 99.7% Sp in non-vaccinated cattle for the Ceditest^® ^(C-ELISA) and 98% Se and 97.2% Sp for the CHEKIT ELISA [39]. Estimates for the C-ELISA give similar ranges of specificity of 95.1% before and 99.4% after heat inactivation in naïve cattle though these drop slightly to 85.3 and 95.7 in vaccinated cattle [40]. A more recent intensive validation exercise has produced estimates of Sp of 97.3% for the I-ELISA, 98.2% for CHEKIT and 97.2% for the Ceditest (the C-ELISA). These are higher than the estimates from this study with the exception of the CHEKIT test [41]. Estimates of diagnostic Se varied with time since infection but were 100% for the I-ELISA more than 100 days post infection compared to the 50% for both the CHEKIT and Ceditest [41]. It is clear from this that the estimates of diagnostic Se depends in part on the time from infection/exposure, the mix of animals sampled and their exposure history. These population specific factors will influence the performance of these test when applied to real populations in the field compared to panels of experimentally derived sera and may in part explain some of the variation in estimates being reported. It is not clear how significant these differences are in practical terms. In addition, it is also important to remember that test parameters are population specific and it is a well recognized problem that diagnostic test can have specificity problems when applied in an African context [42].

## Conclusion

The use of latent class models for the evaluation of diagnostic tests in the absence of a "gold-standard" test constitute a useful alternative or addition to the classic analysis for evaluation of tests, such as antibody tests for FMD. This work has also highlighted the problem of defining "disease" on the basis of serological tests, particularly when serological results have to be interpreted without additional epidemiological information. It also suggests that the C-ELISA and I-ELISA are both highly sensitive tests for use in cattle populations with multiple exposures to different FMD virus serotypes. However, these NSP tests all lack specificity and this may create difficulties in designing sero-surveillance strategies as the number of herds with false positives may overwhelm the available resources to deal with them unless a confirmatory test such as the EITB is also used.

## Methods

### Study design

**Figure 1:**
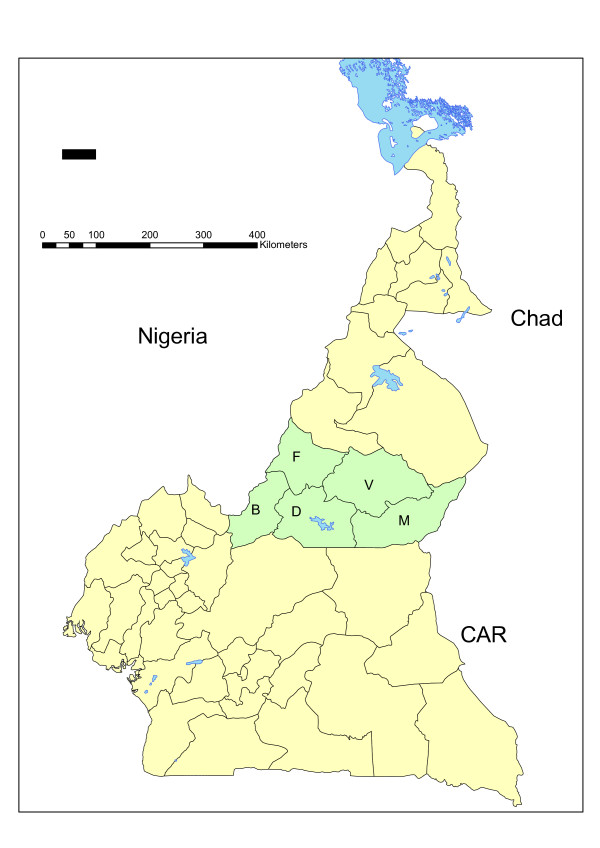
Map of Cameroon showing the Adamawa Province and the five Divisions, Vina (V), Mbere (M), Djerem (D), Mayo Bany0 (B) and Faro et Deo (F).

The study design has been described previously [37]. In brief, the study area was the five administrative Divisions of the 64,000 km^2 ^Adamawa Province of Cameroon (Figure 1). A sample frame of the cattle herds in the area was constructed from the rinderpest vaccination lists held by the local Veterinary Centres. A cross-sectional study design was used with a stratified (by Division), two-stage (by Centre and Herd) random sampling strategy. The program 'Survey Toolbox' [43] calculated a two-stage sample of 54 centres and three herds per centre, allowing for a 10% non-response rate (see Bronsvoort et al. [37] for sample size calculation). The within herd sample strategy was based on a limit of detection calculation [44] and assumed a perfect test and a within herd seroprevalence of 50%. For an average herd of 70 animals with a 95% probability of finding at least 1 positive animal in the sample, a sample size of five is needed. Using random number tables, five juvenile (8–24 month old) and five adult (>24 months old) cattle were cast, examined for lesions and a serum sample taken from each animal. Blood samples were centrifuged at 1100 g for 10 minutes in the field using a 'Mobilespin' 12 V field centrifuge (Vulcon Technologies) or a hand crank centrifuge (OFI Testing Equipment, Inc.) and approximately 3.5 ml of serum was aliquoted into 2 × 1.8 ml cryovials (Nunc). The serum was kept at 4°C in a portable gas fridge until they could be frozen and stored at -20°C. The sera were carried to the UK on dry ice and stored at -20°C at the FMDV World Reference Laboratory, Pirbright, UK (WRL). The herds were presumed to be unvaccinated since no government licenses have been issued to import vaccines into the country and no herdsman reported using an FMDV vaccine. In addition, it is unlikely private herdsmen would be vaccinating independently since the vaccine is very expensive. The field study was conducted between April and October 2000. The diagnostic testing was carried out as described below and each laboratory tested the samples blind without knowing any previous test results.

### Diagnostic tests

#### CHEKIT™-3ABC-FMD ELISA

The CHEKIT-ELISA was used according to the manufacturer's 2002 instructions. Briefly, the serum was diluted 1/100 and added in duplicate to the wells of a 96 well microtitre plate pre-coated with the vector expressed viral 3ABC antigen. Antibodies specific to 3ABC were bound to the antigen forming an antigen/antibody complex on the plate surface. Unbound antibody was washed away. A horseradish peroxidase labeled guinea pig anti-bovine IgG conjugate was added which bound to any antibody/antigen complexes. Unbound conjugate was removed by washing and the chromagen substrate added. The degree of color that developed was proportional to the amount of antibody complexed on the plate surface and read at 405 nm with a spectrophotometer. The final reading for the sample was calculated as follows using the mean of each pair of samples and the median of the 4 positive and 4 negative control sera supplied with the kit and measured on each plate:

value%=ODsample−ODnegODpos−ODneg×100
 MathType@MTEF@5@5@+=feaafiart1ev1aaatCvAUfKttLearuWrP9MDH5MBPbIqV92AaeXatLxBI9gBaebbnrfifHhDYfgasaacH8akY=wiFfYdH8Gipec8Eeeu0xXdbba9frFj0=OqFfea0dXdd9vqai=hGuQ8kuc9pgc9s8qqaq=dirpe0xb9q8qiLsFr0=vr0=vr0dc8meaabaqaciaacaGaaeqabaqabeGadaaakeaacqWG2bGDcqWGHbqycqWGSbaBcqWG1bqDcqWGLbqzcqGGLaqjcqGH9aqpdaWcaaqaaiabd+eapjabdseaenaaBaaaleaacqWGZbWCcqWGHbqycqWGTbqBcqWGWbaCcqWGSbaBcqWGLbqzaeqaaOGaeyOeI0Iaem4ta8Kaemiraq0aaSbaaSqaaiabd6gaUjabdwgaLjabdEgaNbqabaaakeaacqWGpbWtcqWGebardaWgaaWcbaGaemiCaaNaem4Ba8Maem4CamhabeaakiabgkHiTiabd+eapjabdseaenaaBaaaleaacqWGUbGBcqWGLbqzcqWGNbWzaeqaaaaakiabgEna0kabigdaXiabicdaWiabicdaWaaa@5A87@

The manufacturers recommend interpretation was: <20% is negative, 20–30% is ambiguous and >30% is positive. These tests were carried out in IAH, Pirbright, UK in 2002.

#### FMD-3ABC blocking ELISA

The C-ELISA was performed as described previously [23]. Briefly, the original samples from Cameroon were aliquoted and heat treated at 56°C for 2 hours and then shipped to Denmark for testing. Microtiter plates were prepared by capturing 3ABC protein produced in the Baculovirus expression system with a monoclonal antibody (MabL74D5) coated on the plates. Dilutions (1:5) of the sera were added and incubated overnight, washed and the competing antibody, horseradish peroxidase conjugated MabL74D5 added and incubated for a further hour. The plates were washed and the chromagen substrate (TMB H_2_O_2_) was added and incubated for 15 minutes at room temperature at which time the color development was measured at 450 nm. The results were expressed as a percentage of the negative control values from negative sera supplied with the kits as shown:

OD%=ODsampleODmean negative controls×100
 MathType@MTEF@5@5@+=feaafiart1ev1aaatCvAUfKttLearuWrP9MDH5MBPbIqV92AaeXatLxBI9gBaebbnrfifHhDYfgasaacH8akY=wiFfYdH8Gipec8Eeeu0xXdbba9frFj0=OqFfea0dXdd9vqai=hGuQ8kuc9pgc9s8qqaq=dirpe0xb9q8qiLsFr0=vr0=vr0dc8meaabaqaciaacaGaaeqabaqabeGadaaakeaacqWGpbWtcqWGebarcqGGLaqjcqGH9aqpdaWcaaqaaiabd+eapjabdseaenaaBaaaleaacqWGZbWCcqWGHbqycqWGTbqBcqWGWbaCcqWGSbaBcqWGLbqzaeqaaaGcbaGaem4ta8Kaemiraq0aaSbaaSqaaiabd2gaTjabdwgaLjabdggaHjabd6gaUjabbccaGiabd6gaUjabdwgaLjabdEgaNjabdggaHjabdsha0jabdMgaPjabdAha2jabdwgaLjabbccaGiabdogaJjabd+gaVjabd6gaUjabdsha0jabdkhaYjabd+gaVjabdYgaSjabdohaZbqabaaaaOGaey41aqRaeGymaeJaeGimaaJaeGimaadaaa@5FD7@

The recommended cut-off is ≤ 50% for a positive result. These tests were carried out in Denmark in 2002.

#### FMD-PANAFTOSA 3ABC I-ELISA

Aliquots of the heat treated sera were sent to PANAFTOSA for screening using the I-ELISA. This test has been described in detail elsewhere [9,45]. Briefly, this is an indirect ELISA using *E.coli *expressed purified polyprotein 3ABC coating of the plates. Test or reference sera were added in 1:20 dilution in blocking buffer (PBS/0.05% Tween 20/5% non-fat milk/10% equine sera/0.1% *E.coli *537 extract) and incubated for 30 minutes at 37°C. Bound bovine antibodies were detected with rabbit anti-bovine IgG peroxidise conjugate (Sigma Chemical CO.) and 3,3',5,5'-tetramethlybenzidine plus 0.004% (w/v) H_2_O_2 _in phosphate-citrate buffer at pH 5.0. The colour development was stopped after 15 minutes using 2 M H_2_SO_4 _and the OD read at 450 nm. Results were expressed as percentage positivity referred to a positive control sera. Percentage positivity values ≥ 10 were considered positive. Two samples had insufficient sera for completion of the I-ELISA test, so the final data set was for 1,375 animals. These tests were carried out at PANAFTOSA, Brazil in 2004.

### Data sets

The results of the three NSP tests were entered into a database and cross tabulated according to whether all tests were positive (+/+/+), the CHEKIT and C-ELISA were positive and the I-ELISA negative (+/+/-) etc as shown in Table 1 for the all animals and in Table 2 for the sub-population of juveniles (8–24 month old).

### Statistical analysis

Hui & Walter [26] introduced a latent class approach to the evaluation of diagnostic tests in absence of a "gold-standard". The Hui-Walter paradigm for test evaluation in the absence of a "gold-standard" requires the presence of two (or more) tests evaluated in two (or more) populations and furthermore that: the prevalence of the disease is different within each population; the tests have the same properties across populations; and the tests must be conditionally independent given the disease status. Conditional independence given disease status between two tests implies that if the true status of the test subject is known, then knowing the outcome (e.g. positive) of one of the tests will not change our belief in a specific test result (e.g. positive) of the other test.

In Toft *et. al. *[46] the original maximum likelihood formulation was compared to a Bayesian model. For sample sizes such as those in the present study the Bayesian model is preferable. The Bayesian version of the Hui-Walter model assumes that for the *i*th subpopulation the counts (**O**_**i**_) of the different combinations of test results, e.g. +/+/+, +/+/-, +/-/+, etc for three tests, follow a multinomial distribution:

**O**_**i**_|Se_j_, Sp_j_, p_i _~ Multinominal(**Pr**_**i**_, n_i_) for i = 1,2,..., S and j = 1,2,..., T

where S is the number of subpopulations and T is the number of tests and **Pr**_**i **_is a vector of probabilities of observing the individual combinations of test results. Conditioning on the (latent) disease status, these probabilities can be specified using Se and Sp of the tests and the prevalence (p) of the subpopulations. As an example, for three tests the probability of observing all three tests positive in the *i*th subpopulation is given as:

Pr(T_1_+, T_2_+, T_3_+) = Pr(T_1_+, T_2_+, T_3_+|D+)Pr(D+) + Pr(T_1_+, T_2_+, T_3_+|D-)Pr(D-)     Eq.1

= Pr(T_1_+|D+) Pr(T_2_+|D+) Pr(T_3_+|D+)Pr(D+)

+ Pr(T_1_+|D-) Pr(T_2_+|D-) Pr(T_3_+|D-)Pr(D-)     Eq.2

= Se_1 _Se_2 _Se_3 _p_i _+ (1-Sp_1_) (1-Sp_2_) (1-Sp_3_)(1-p_i_)     Eq.3

where the first transformation (Eq.1) uses conditioning on the disease status, the second (Eq.2) utilizes the assumption of conditional independence between tests given disease status and the third (Eq.3) merely renames the parameters using more familiar terms. The other seven probabilities for the three test scenarios may be similarly derived.

In a Bayesian analysis all parameters are given distributions. Hence, prior distributions for the test properties and the prevalence within the subpopulations must be specified. For this analysis, we did not want to utilize potential prior information about the tests and prevalence, thus we chose to use uninformative priors in the shape of uniform distributions on the interval between zero and one, modelled using the Beta(1,1) distribution.

The model was implemented in WinBUGS [47], which is a general purpose modelling tool which uses Markov Chain Monte Carlo (MCMC) methods for Bayesian inference. Put simply, MCMC are a method for sampling from the posterior distribution of interest (the Markov Chain element) and subsequently calculating the relevant measures, e.g. means, medians and standard deviations of the parameters (the Monte Carlo element). To ensure that the sample is obtained from the distribution of interest, the first part of the Markov Chain, the so-called burn-in, is discarded and the subsequent samples are used for inference. However, prior to using the samples for inference, the convergence of the sample chain must be assessed. For this analysis, the first 5,000 iterations were discarded as a burn-in and every tenth of the following 50,000 iterations were kept for posterior inference (the chain was thinned, to reduce auto-correlation between samples). Convergence of the chain after the initial burn-in was assessed by visual inspection of the time-series plots for the parameters as well as Gelman-Rubin diagnostic plots using three sample chains with different starting values [48].

The samples were used for inference by calculating posterior means and posterior 95% credibility intervals (PCI) for the Se, Sp and p. The PCI is the central 95% of the probability distribution for a parameter, i.e. endpoints are the 2.5% and 97.5% percentiles, respectively. There were no discernible differences between posterior means and medians; hence the means were reported for purposes of discussion, while the 95% PCI should be seen as the primary results.

## Authors' contributions

BB Study design, field work, data analysis, preparation of manuscript

NT Data analysis and modelling, preparation of manuscript

IB Screening of sera using I-ELISA, preparation of manuscript

KS Screening of sera using C-ELISA, preparation of manuscript

JA Screening of sera using CHEKIT, preparation of manuscript

VM Screening of sera using I-ELISA, preparation of manuscript

VN Study design, field work, preparation of manuscript

KM Study design, field work, preparation of manuscript

## Figures and Tables

**Table 1:** Cross-tabulated results for the CHEKIT, C-ELISA and I-ELISA NSP tests.

	Test combination (Chekit CELISA I-ELISA)								
Province	+/+/+	+/+/-	+/-/+	+/-/-	-/+/+	-/+/-	-/-/+	-/-/-	Total (n)
Vina	78	4	1	3	166	23	26	173	474
Mbere	44	0	2	1	54	18	8	96	223
Djerem	24	0	1	0	52	11	20	145	253
Mayo Banyo	31	0	2	1	103	9	24	105	275
Faro et Deo	29	1	0	0	42	9	7	62	150

Cross-tabulated results for the CHEKIT, C-ELISA and I-ELISA NSP tests in the full population stratified by the five administrative Divisions in the Adamawa Province of Cameroon (n = 1,375).

**Table 2:** Cross-tabulated results for the CHEKIT, C-ELISA and I-ELISA NSP tests.

	Test combination (Chekit C-ELISA I-ELISA)								
Province	+/+/+	+/+/-	+/-/+	+/-/-	-/+/+	-/+/-	-/-/+	-/-/-	Total (n)
Vina	20	3	1	1	51	10	15	128	229
Mbere	3	0	0	1	19	9	4	64	100
Djerem	8	0	0	0	10	1	7	84	110
Mayo Banyo	3	0	2	1	33	5	8	83	135
Faro et Deo	11	0	0	0	15	2	1	46	75

Cross-tabulated results for the CHEKIT, C-ELISA and I-ELISA NSP tests in the sub data set of juvenile (8–24 month old) animals, stratified by the five administrative Divisions in the Adamawa Province of Cameroon (n = 649).

**Table 3:** Latent class estimates (posterior mean and 95% credibility intervals (PCI)) of test Se and Sp and Divisional seroprevalence for the full data set (adults and juveniles) using all three tests and pairs of tests in combination

	All three tests		I-ELISA+C-ELISA		I-ELISA+CHEKIT		CHEKIT+C-ELISA	
Parameter	Mean	95% PCI	Mean	95% PCI	Mean	95% PCI	mean	95% PCI
Se_CHEKIT_	**33.2**	(29.6; 37.0)	**-**	-	**34.2**	(28.0; 45.1)	**36.0**	(29.1; 47.0)
Sp_CHEKIT_	**99.0**	(98.1; 99.1)	**-**	-	**99.3**	(98.0; 100.0)	**99.1**	(97.7; 100.0)
Se_C-ELISA_	**96.9**	(94.0; 99.1)	**95.0**	(88.2; 99.7)	**-**	-	**96.6**	(92.7; 99.8)
Sp_C-ELISA_	**90.9**	(87.9; 93.8)	**94.9**	(88.9; 99.7)	**-**	-	**86.4**	(72.3; 98.8)
Se_I-ELISA_	**97.1**	(94.4; 99.1)	**93.3**	(88.5; 99.1)	**96.4**	(92.7; 99.6)	**-**	-
Sp_I-ELISA_	**88.8**	(85.8; 92.0)	**90.8**	(85.6; 97.9)	**88.2**	(72.3; 99.3)	**-**	-

p_vina_	**54.1**	(49.1; 59.1)	**57.4**	(51.1; 63.6)	**53.5**	(41.1; 61.8)	**51.7**	(39.5; 60.7)
p_mbere_	**46.3**	(39.4; 53.3)	**50.2**	(41.5; 58.5)	**44.5**	(33.7; 53.5)	**47.2**	(35.5; 57.1)
p_djerem_	**30.1**	(25.1; 37.0)	**34.0**	(26.7; 42.1)	**30.9**	(17.8; 41.8)	**25.8**	(14.5; 37.0)
p_banyo_	**50.9**	(44.5; 57.2)	**54.3**	(46.6; 62.5)	**52.5**	(36.6; 62.8)	**44.5**	(29.3; 56.2)
p_faro_	**49.4**	(41.4; 57.5)	**53.0**	(43.5; 61.8)	**48.0**	(35.2; 58.9)	**48.4**	(35.7; 59.9)

**Table 4:** Latent class estimates (posterior mean and 95% credibility intervals (PCI)) of test Se and Sp and Divisional seroprevalence for the sub-data set (juveniles only) using all three tests and pairs of tests in combination

	All three tests		I-ELISA+C-ELISA		I-ELISA+CHEKIT		CHEKIT+C-ELISA	
Parameter	Mean	95% PCI	Mean	95% PCI	Mean	95% PCI	mean	95% PCI
Se_CHEKIT_	**26.1**	(20.1; 32.6)	**-**	-	**43.2**	(22.3; 82.0)	**47.7**	(22.5; 90.0)
Sp_CHEKIT_	**99.1**	(97.9; 99.8)	**-**	-	**99.2**	(97.7;100.0)	**99.0**	(97.5;100.0)
Se_C-ELISA_	**92.6**	(84.5; 98.3)	**90.4**	(80.0; 99.5)	**-**	-	**93.6**	(82.5; 99.8)
Sp_C-ELISA_	**96.1**	(92.8; 99.4)	**97.0**	(92.4; 100.0)	**-**	-	**83.2**	(72.9; 98.5)
Se_I-ELISA_	**92.3**	(84.9; 98.0)	**90.5**	(82.7; 99.1)	**91.6**	(80.6; 99.4)	**-**	-
Sp_I-ELISA_	**93.7**	(90.2; 97.7)	**94.9**	(90.0; 99.6)	**82.4**	(72.0; 97.5)	**-**	-

p_vina_	**38.2**	(31.1; 45.9)	**39.8**	(31.1; 48.6)	**28.3**	(12.5; 44.5)	**25.8**	(11.3; 43.0)
p_mbere_	**26.7**	(17.4; 37.9)	**29.5**	(18.6; 41.2)	**11.8**	(2.1; 29.1)	**14.7**	(2.0; 36.6)
p_djerem_	**21.0**	(13.6; 29.4)	**22.6**	(14.4; 32.0)	**16.0**	(6.7; 30.3)	**12.9**	(5.4; 23.6)
p_banyo_	**31.5**	(23.1; 40.7)	**32.7**	(23.7; 42.6)	**17.8**	(3.4; 37.9)	**13.9**	(1.5; 35.6)
p_faro_	**36.6**	(26.0; 48.0)	**37.6**	(26.7; 49.2)	**28.1**	(13.8; 27.8)	**28.3**	(12.9; 46.2)
